# Acute Effects of High Intensity, Resistance, or Combined Protocol on the Increase of Level of Neurotrophic Factors in Physically Inactive Overweight Adults: The BrainFit Study

**DOI:** 10.3389/fphys.2018.00741

**Published:** 2018-06-27

**Authors:** María A. Domínguez-Sanchéz, Rosa H. Bustos-Cruz, Gina P. Velasco-Orjuela, Andrea P. Quintero, Alejandra Tordecilla-Sanders, Jorge E. Correa-Bautista, Héctor R. Triana-Reina, Antonio García-Hermoso, Katherine González-Ruíz, Carlos A. Peña-Guzmán, Enrique Hernández, Jhonatan C. Peña-Ibagon, Luis A. Téllez-T, Mikel Izquierdo, Robinson Ramírez-Vélez

**Affiliations:** ^1^Grupo de Investigación Movimiento Corporal Humano, Facultad de Enfermería y Rehabilitación, Universidad de La Sabana, Chía, Colombia; ^2^Evidence-Based Therapeutic Group, Clinical Pharmacology, Universidad de La Sabana, Bogotá, Colombia; ^3^Centro de Estudios en Medición de la Actividad Física, Escuela de Medicina y Ciencias de la Salud, Universidad del Rosario, Bogotá, Colombia; ^4^Grupo GICAEDS, Programa de Cultura Física, Deporte y Recreación, Universidad Santo Tomás, Bogotá, Colombia; ^5^Laboratorio de Ciencias de la Actividad Física, el Deporte y la Salud, Universidad de Santiago de Chile, Santiago, Chile; ^6^Grupo de Ejercicio Físico y Deportes, Facultad de Salud, Programa de Fisioterapia, Universidad Manuela Beltrán, Bogotá, Colombia; ^7^Facultad de Ingeniería Ambiental, Grupo de Investigación INAM-USTA Universidad Santo Tomás, Bogotá, Colombia; ^8^Department of Health Sciences, Public University of Navarra, Navarrabiomed, CIBER of Frailty and Healthy Aging (CIBERFES) Instituto de Salud Carlos III, Pamplona, Spain

**Keywords:** neurotrophic factors, exercise, obesity, inactivity, plasticity

## Abstract

The purpose of this study was to compare the neurotrophic factor response following one session of high-intensity exercise, resistance training or both in a cohort of physically inactive overweight adults aged 18–30 years old. A randomized, parallel-group clinical trial of 51 men (23.6 ± 3.5 years; 83.5 ± 7.8 kg; 28.0 ± 1.9 kg/m^2^) who are physically inactive (i.e., < 150 min of moderate-intensity exercise per week or IPAQ score of <600 MET min/week for >6 months) and are either abdominally obese (waist circumference ≥90 cm) or have a body mass index, BMI ≥25 and ≤ 30 kg/m^2^ were randomized to the following four exercise protocols: high-intensity exercise (4 × 4 min intervals at 85–95% maximum heart rate [HRmax] interspersed with 4 min of recovery at 75–85% HRmax) (*n* = 14), resistance training (12–15 repetitions per set, at 50–70% of one repetition maximum with 60 s of recovery) (*n* = 12), combined high-intensity and resistance exercise (*n* = 13), or non-exercising control (*n* = 12). The plasma levels of neurotrophin-3 (NT-3), neurotrophin-4 (also known as neurotrophin 4/5; NT-4 or NT-4/5), and brain-derived neurotrophic factor (BDNF) were determined before (pre-exercise) and 1-min post-exercise for each protocol session. Resistance training induced significant increases in NT-3 (+39.6 ng/mL [95% CI, 2.5–76.6; *p* = 0.004], and NT-4/5 (+1.3 ng/mL [95% CI, 0.3–2.3; *p* = 0.014]), respectively. Additionally, combined training results in favorable effects on BDNF (+22.0, 95% CI, 2.6–41.5; *p* = 0.029) and NT-3 (+32.9 ng/mL [95% CI, 12.3–53.4; *p* = 0.004]), respectively. The regression analysis revealed a significant positive relationship between changes in BDNF levels and changes in NT-4/5 levels from baseline to immediate post-exercise in the combined training group (*R*^2^ = 0.345, *p* = 0.034) but not the other intervention groups. The findings indicate that acute resistance training and combined exercise increase neurotrophic factors in physically inactive overweight adults. Further studies are required to determine the biological importance of changes in neurotrophic responses in overweight men and chronic effects of these exercise protocols.

Trial Registration: ClinicalTrials.gov, NCT02915913 (Date: September 22, 2016).

## Introduction

The neurotrophin family of growth factors, comprised of nerve growth factors, brain-derived neurotrophic factor (BDNF), neurotrophin-3 (NT-3), and neurotrophin-4/5 (NT-4/5), all of which were originally defined by their ability to support the survival, development, and function of neurons (Dechant and Neumann, 2003). Moreover, a metabotrophic role of nerve growth factor and BDNF has recently been implicated in the pathogenesis of metabolic related disorders, sensation, and energy homeostasis (Hristova, 2013). Likewise, the BDNF/tyrosine kinase receptor signaling pathway controls feeding and metabolism, and its dysfunction leads to severe obesity or subclinical inflammatory diseases such as metabolic syndrome, insulin resistance, and type 2 diabetes (Tonra et al., 1999; Nonomura et al., 2001; Cai et al., 2006; Rosenthal and Lin, 2014; Wei et al., 2017).

In contrast to metabolic related disorders-induced BDNF changes, Mercader et al. (2008) analyzed an NT-4/5 knockout mouse model and showed that this mice displays reduced food satiation and hyperphagic behavior resulting in an obese mouse when fed *ad libitum*. Furthermore, the infusion of NT-4/5 into the third ventricle of the brain reversed the obese phenotype, which suggests that NT-4/5 is involved in the activation of signaling cascades in the hypothalamic nuclei; these cascades are responsible for the control of food intake and energy expenditure.

Interestingly, physical exercise (particularly moderate to vigorous training) is an effective strategy to combat metabolic disorders due to its ability to influence body composition and some biomarkers, such as cholesterol and insulin resistance (Gibala and McGee, 2008; Buch et al., 2017). During high-intensity exercise (>80% of maximal oxygen intake), significant increases in neurotrophic factor have been observed (Marston et al., 2017). In this line, Saucedo-Marquez et al. (2015) showed that after a 20-min single session of either a continuous exercise protocol (at 70% of maximal work rate) or an high-intensity interval training (HIIT) protocol (at 90% of maximal work rate for periods of 1 min alternating with 1 min of rest), the BDNF levels were increased compared to the levels at rest. Tonoli et al. (2015) showed that a combined session of HIIT and continuous exercise increased serum BDNF and IGF-I levels in 10 participants with type 1 diabetes. Verbickas et al. (2017) showed that serum BDNF levels were decreased at 1–24 h after 200 drop jumps. Afzalpour et al. (2015) compared the effects of 6 weeks of HIIT and continuous training regimens on the levels of BDNF, glial cell line-derived neurotrophic factor (GDNF) and tumor necrosis factor alpha (TNF-α) in the rat brain. These authors showed that both HIIT and continuous training regimens significantly increased BDNF and GDNF concentrations with larger increases following HIIT than continuous training.

Other studies report increases in peripheral BDNF following acute resistance training (RT) (Yarrow et al., 2010; Marston et al., 2017), whereas others report no change (Correia et al., 2010; Goekint et al., 2010). These discrepancies can likely be explained by the wide range of characteristics of the participants (age, health status, diseases, etc.), study duration, intervention exercise program (i.e., jumping, treadmill, and/or elliptical, weight machines), and the extent of change in body composition across these studies. There are also reports of exercise-induced changes in NT-3 (Gómez-Pinilla et al., 2001; Johnson and Mitchell, 2003) and NT-4/5 (Chung et al., 2013) expression following focal cerebral ischemia or spinal cord injury in rats. However, there are no reports of exercise-induced changes in plasma NT-3 or NT-4/5 levels in humans. There is considerable information available concerning the endocrine response in lean individuals as a point of reference for future research using an inactive/overweight model.

Therefore, the aim of the present study was to compare the acute responses of the neurotrophic factors BDNF, NT-3, and NT-4/5 prior to and after one session of exercise comprising HIIT, RT, or both in a cohort of physically inactive overweight adults aged 18–30 years old. It was hypothesized that the combined training protocol would induce the highest metabolic perturbations and therefore the highest hormonal responses to a greater extent than RT group and HIIT alone.

## Methods

### Study design and participants

The BrainFit Study is a single blind, randomized controlled 2 × 2 factorial trial (ClinicalTrials.gov ID: NCT02915913). The study was approved by the Medical Research Ethics Committee of The Universidad Nacional de Colombia (Code N° 018-223-16). Fifty-one men (aged 18–30 years), who were inactive (according to the International Physical Activity Questionnaire, IPAQ <150 min of moderate-intensity exercise per week or IPAQ score of <600 MET min/week for greater than 6 months), had abdominal obesity (defined as waist circumference [WC] ≥ 90 cm) or excess weight (defined as BMI ≥25 and ≤30 kg/m^2^) were recruited from Bogota, Colombia. The latest International Diabetes Federation/National Heart, Lung, and Blood Institute/American Heart Association (IDF/NHLBI/AHA-2009) consensus stated that WC was measured according to Country/Specific values which, for Latin Americans, were set to be equal to South Asian parameters, specifically WC ≥90 cm for males (Alberti et al., 2009). Subjects were recruited between September 1, 2016, and June 30, 2017. Healthy and inactive men who were currently not taking drugs, tobacco, or any other medications were included in this study. A validated questionnaire, the “FANTASTIC” lifestyle (Ramírez-Vélez and Agredo, 2012), was used to collect comprehensive information about substance use via a personal interview with participants. At the beginning of every session, quality of sleep, smoking, alcohol, and caffeine consumption, before the experiment were documented. The final follow-up visit was in July 2017. Inclusion and exclusion criteria are provided in Table 1.

**Table 1:** Inclusion/exclusion criteria.

**Inclusion criteria**	**Exclusion criteria**
a. Inactive: no participation in supervised exercise more than once a week for the previous 6 months, according to the IPAQ score of <600 MET min/week	a. Systemic infections
b. Central obesity: waist circumference ≥90 cm or excess weight: body mass index ≥25 and ≤30 kg/m^2^	b. Weight loss or gain of >10% of body weight in the past 6 months for any reason
c. Interested in improving cardiovascular health and physical fitness	c. Currently taking medication that suppresses or stimulates appetite
d. Written informed consent	d. Uncontrolled hypertension: systolic blood pressure 160 mmHg or diastolic blood pressure 95 mmHg on treatment
e. Interested in improving cardiovascular health and physical fitness	e. Gastrointestinal disease, including self-reported chronic hepatitis or cirrhosis, any episode of alcoholic hepatitis or alcoholic pancreatitis within past year, inflammatory bowel disease requiring treatment in the past year, recent or abdominal surgery (e.g., gastrostomy)
	f. Asthma
	g. Diagnosed diabetes (type 1 or 2), fasting impaired glucose tolerance (blood glucose 118 mg/dL), or use of any anti-diabetic medications
	h. Currently taking antidepressant, steroid, or thyroid medication, unless dosage instable (no change for 6 months)
	i. Current exerciser (>30 min organized exercise per week)
	j. Indication of unsuitability of current health for exercise protocol (Physical Activity Readiness. Questionnaire, PARQ)
	k. Any other conditions which, in opinion of the investigators, would adversely affect the conduct of the trial

### Recruitment

Consecutive males with either abdominal obesity or excess weight were recruited from different educational institutions (Universidad Nacional, CEMA-Universidad del Rosario, Universidad Santo Tomás, Universidad Manuela Beltrán, and Universidad de la Sabana) that receive referrals from both medical consultants and biomedical practitioners in the capital district of Bogotá and the Cundinamarca Department in the Andean region (Ramírez-Vélez et al., 2016). Subjects who are interested in participating were provided additional information and underwent the following procedures: (i) initially screened for pre-participation exercise using a cardiovascular and musculoskeletal checklist; (ii) baseline testing; (iii) a single isocaloric acute training protocol; and (iv) post-training testing. All participants were informed of the purpose and risk of the study before signing an informed consent form.

### Blinding and randomization methods

Randomization into the four study arms was performed by CEMA at the University of Rosario, Bogotá, Colombia, using block randomization with a block size of four. After completing the baseline measurements, eligible participants were randomly assigned to either the control or one of the exercise training groups. Participants were randomly allocated using a computer-generated randomization code created before the data collection by an investigator not involved in the assessment or treatment of the participants. These procedures are also detailed in the study operations manual. Assessors were also blinded to each participant's treatment allocation.

### Preliminary analysis

Each of the volunteers participated in three randomized trials (HIIT, RT, HIIT+RT, and control [no exercise]), and the starting trial was randomized. At 48 h after the start of the training period, the 1RM was measured for six different exercises: bicep screw curl, triceps extension, dumbbell side lateral raise, military press, dumbbell squat, and dumbbell front lunge which was implemented based on similar procedures (Ramírez-Vélez et al., 2016). The 1RM was performed in six resistance exercises and was conducted between 09:00 and 11:00 a.m.; the highest load of three attempts per exercise were reported. Total muscle strength was calculated as the sum of the six exercises. The 50–70% value of the 1RM was used to determine the work load during the single sessions for the experimental group.

Maximal oxygen intake (VO_2_max) of inactive subjects was determined 24 h before acute intervention using a maximum treadmill exercise test (Precor TRM 885, Italy). Subjects completed an incremental maximal oxygen uptake test on a treadmill ergometer. A metabolic cart with an on-line gas collection system (COSMED K5 portable metabolic system, Rome, Italy) was used to measure VO_2_max and carbon dioxide production data, and HR was continuously monitored with a HR monitor (A3, Polar Elector OY, Finland).

### Exercise training protocol

The current exercise protocol was based on data derived from the Cardiometabolic HIIT-RT Study (ClinicalTrials.gov ID: NCT02715063). Details of methods had been previously published elsewhere (Ramírez-Vélez et al., 2016), however, the most relevant information is briefly described below.

**Figure 1:**
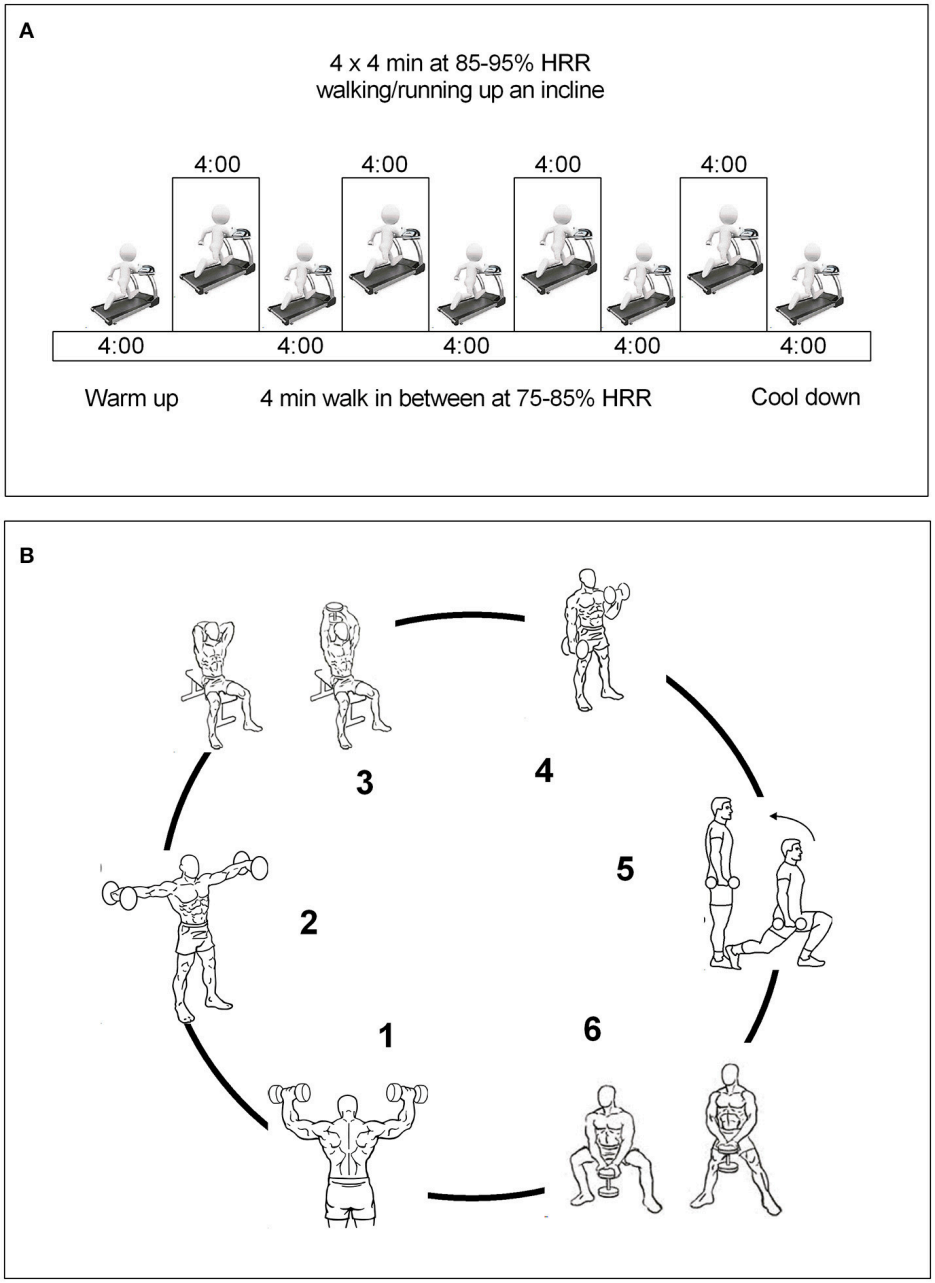
Run-in training interventions. **(A)**, HIIT group; **(B)**, RT group. Combined training group were received both the HIIT and RT protocols as described above.

1. *Control group*: Without exercise training.
2. *High-intensity interval training (HIIT) group:* All HIIT sessions were preceded with a 5-min warm-up and ended with a 4-min cooldown at a 65% heart rate maximal until the subject expended between 400 and 500 kcal. The HIIT protocol consisted of four bouts of 4-min intervals at 85–95% HR maximal interspersed with 4 min of active recovery at 75–85% HR maximal. Participants in the HIIT groups were instructed to reach their target HR for each interval within the first 2 min of the 4 min interval. We calculated the training energy expenditure with the consensus public health recommendations from the World Health Organization (WHO, 2004) and the US Department of Health and Human Services (Physical Activity Guidelines Advisory Committee, 2008). Heart-rate monitors (A3, Polar Elector OY, Finland) were used to adjust workload to achieve the target heart rate. In addition, a rating of perceived exertion was also measured during each exercise session (15–17 during high intensity and 11–13 during recovery), (Figure 1A).
3. *Resistance training (RT) group:* The RT session was initiated with ≈12–15 repetitions per set of six exercises that targeted all the major muscle groups at high intensity. A 60-s recovery was permitted as many times as needed according to subject's weight until the subject expended between 400 and 500 kcal at 50–70% one repetition maximum (1RM). The RT included both upper and lower body large-muscle exercises using weight machines (bicep screw curl, triceps extension, dumbbell side lateral raise, military press, dumbbell squat, and dumbbell front) (Ramírez-Vélez et al., 2016). Our exercise structure, is similar to utilized by Church et al. (2016) is considered moderate to vigorous intense and therefore it would be expected resulted in increased levels of peripheral BDNF, (Figure 1B).
4. *Combined training (HIIT*+*RT) group:* This group underwent both the HIIT and RT protocols, as described above, in that order. Therefore, the energy expenditure associated with the physical training prescribed for the vigorous intensity group was ≈400–500 kcal/session (Ramírez-Vélez et al., 2016).

### Training intensity and energy expenditure during the exercise session

In terms of exercise intensity, the actual intensity values were reported as the mean of HR measured in the HIIT and combined groups and as the average value of workload and repetitions determined in the acute session in RT group. The intensity of the HIIT or HIIT+RT group was based on the percentage of each individual's HRmax derived from a maximal treadmill test. The exercise training was 100% supervised. Research staff monitored and recorded compliance with target HR and energy expenditure during the sessions. Energy expenditure was estimated during exercise via indirect calorimetry using a COSMED *K*^5^ portable metabolic system (Rome, Italy) assuming a non-protein respiratory exchange ratio (Graf et al., 2013). It was expected that the gradual increase in total energy expenditure would minimize fatigue, soreness, injuries, and attrition.

### Blood draws and analysis

Participants arrived at the CEMA laboratory between 6:00 and 9:00 following a 10–12 h overnight fast. Participants were reminded to maintain standardized conditions (i.e., hydrated state and abstaining from caffeine and alcohol consumption for 36 h). Blood samples were drawn into a tube containing ~1.8 mg EDTA-K^3^ per mL blood for plasma measurements and a tube with polymer gel for serum measurements (Vacutainer, Becton Dickinson and Company 2017). Samples were handled according to Clinical Laboratory Improvement Amendments, which must be followed in order to achieve valid test results for accurate diagnoses (Centers for Medicare Medicaid Service, 2005). Finally, both samples were stored at −80°C for the analysis of plasma biomarkers of neuronal function using surface plasmon resonance (SPR) biosensors.

### Surface plasmon resonance (SPR) biosensors

SPR allows real-time monitoring of NT-3, NT-4/5, and BDNF (R&D Systems, Minnesota, USA) (Bustos et al., 2014). In a typical experiment, the signal reflection, measured at a fixed angular position, is evaluated as a function of time. The real-time strategy of quantification involved specific antibodies to the target proteins. All experiments were carried out at 25°C using a SPR Biacore 2000 automatic (Biacore, Uppsala, Sweden). HBS-EB buffer was used as running buffer at 5–60 μg/mL flow. Anti-neurotrophin-3, anti-neurotrophin-4 and anti-BDNF antibodies were immobilized onto the CM5 sensor surface at a concentration ranging from 10 to 50 μg/mL. Before immobilization of the antibodies, the sensor surface was activated via an amino-coupling chemistry kit K AN-50 Coupling Kit (GE Healthcare, Uppsala, Sweden). For the immobilization steps, the basic principles of assays using SPR were applied, such as preconcentration and binding assay with the analyte. Excess activated carboxyl groups were blocked with 1 M ethanolamine hydrochloride (pH 9.5). A reference or control flow cell containing 50 μg/mL bovine serum albumin (Sigma-Aldrich, Saint Louis, MO) at pH 5.0 was used to subtract the instrument's systematic noise and drift (i.e., background response). After each injection of the analyte and standard samples to the binding assay, an optimal regeneration solution was injected as described in previous studies (Andersson et al., 1999; Kuroki and Maenaka, 2011). The percentage of surface regeneration was estimated using the following equation: [1 – (Rreg/Ro) × 100]. The complete regeneration was higher than 50%. Standard curves were constructed by diluting standards of recombinant NT-3, NT-4, and BDNF protein (Bustos et al., 2014). A range of 20–5,000 ng/ mL was used to obtain the curve (Bustos et al., 2014). The manufacturer declares intra- and inter-assay coefficients of variation of <10%.

### Body composition and anthropometric measures

Body fat mass was determined with a multifrequency bioelectrical impedance analyser (BIA) using tetrapolar whole body impedance (Model Seca® mBCA 514 Medical Body Composition Analyzer, Hamburg, Germany). A detailed description of the BIA technique can be found in a previous study (Rodríguez-Rodríguez et al., 2016). The intra-observer technical error (% reliability) of the measurements was 95%.

The values of height and body mass were recorded in meters to the nearest 0.1 cm and 0.1 kg with an electronic height (Seca® 274, Hamburg, Germany) and weight scale (Model Seca® mBCA 514 Medical Body Composition Analyzer, Hamburg, Germany). BMI was calculated with the following formula: BMI = body weight (kg)/height squared (m^2^) (WHO, 2000). Waist circumference (WC) was also measured to the nearest 1 mm with a flexible steel tape measure (Lufkin W606PM®, Parsippany, New Jersey, USA) placed midway between the lowest rib and the iliac crest while participants were in a standing position at the end of an exhalation in accordance with the International Society for the Advancement of Kinanthropometry guidelines (Marfell-Jones et al., 2012). The technical error of measurement values was <2% for all anthropometric variables.

### Statistical analysis

To calculate the required sample size, we used the formula for the comparison of two means: *n* = [A + B]^2^ × 2 × *SD*^2^/DIFF^2^, where n = the sample size required in each group, *SD* = standard deviation of the outcome variable and DIFF = size of the desired difference between groups. A and B depend on the desired significance level and desired power, respectively. Using estimates obtained from the literature (Saucedo-Marquez et al., 2015; Tonoli et al., 2015), a sample size of eight subjects in each group was needed to reach a power of 80% to detect a difference in means in the relative change of BDNF (10%Δ after 5 min of HIIT training, assuming an SD of 588 pg/mL using a two-sample *t*-test with a 0.05 two-sided significance level). Assuming a drop-out rate of 20%, the total minimal sample size has been increased to 12 subjects for each group. We believe this sample size is feasible and realistic based on other experience in experimental studies (Gómez-Pinilla et al., 2001; Johnson and Mitchell, 2003; Krabbe et al., 2007; Rasmussen et al., 2009; Seifert et al., 2010; Chung et al., 2013; Saucedo-Marquez et al., 2015).

Comparisons between differences of mean values of normally distributed variables between groups of exercise were tested using linear mixed-effects modeling for repeated measures over time using BDNF, NT-3, and NT-4 as the dependent variable and effects for time, group (HIT, RT, HIIT+RT, or control group), and time by group interaction including their baseline measurement and VO_2_max the intervention time as co-variables, following the same procedures as in the intent-to-treat analyses. The significance level adopted to reject the null hypothesis was *p* < 0.05, and effect size for paired comparisons was reported as η^2^ partial throughout; these values are interpreted as small (≥0.02), moderate (≥0.13), and large (≥0.26). Regression analyses were used to examine the relationship between changes in BDNF and other neurotrophic factors. Finally, goodness-of-fit tests were performed to determine whether the proportion of participants who showed higher final neurotrophic levels in each protocol was equal. Parametric datasets are summarized in text as the mean (*SD*), standard error of the mean (SEM) and 95% CI. All analyses were performed using the SPSS software package (Version 22, IBM, New York, USA).

## Results

**Figure 2:**
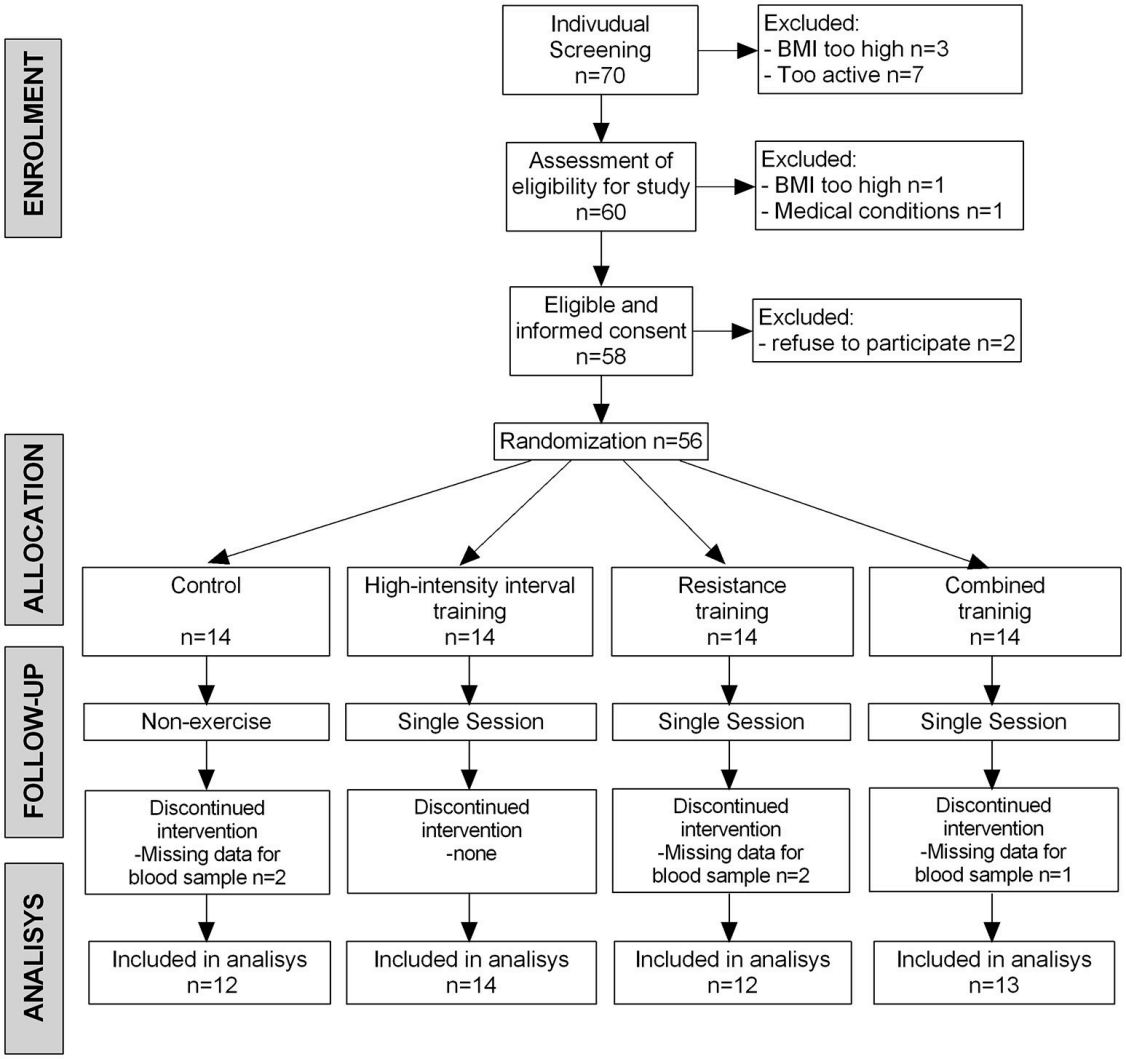
The BrainFit Trial Flow Diagram. Those whose blood samples (*n* = 5) were technically inadequate and not analyzed. BMI, body mass index.

Of the 70 participants who entered the run-in phase, 56 (80%) were randomized. Reasons for pre-randomization exclusion included excessive BMI, refusal to participate, or a medical condition (Figure 2). Five additional participants (2 from the control group, 2 from the RT group, and 1 from the combined group) were excluded from the blood sample analyses because their serum was technically inadequate.

Descriptive statistics for each sex group are shown in Table 2. No significant intergroup baseline differences were observed.

**Table 2:** Baseline participant characteristics by group training.

**Characteristics**	**Group training**			
	**Control (*n* = 12)**	**HIIT (*n* = 14)**	**RT (*n* = 12)**	**HIIT+RT (*n* = 13)**
Age, y	24.7 (3.4)	24.5 (3.7)	22.8 (3.1)	22.2 (3.4)
Weight, kg	88.6 (8.9)	81.7 (6.7)	83.9 (7.4)	80.6 (6.7)
Height, m	1.75 (0.05)	1.72 (0.05)	1.68 (0.18)	1.69 (0.05)
BMI, kg/m^2^	28.7 (2.0)	27.4 (1.7)	27.8 (1.3)	28.1 (1.2)
Waist circumference, cm	97.9 (6.3)	95.3 (4.9)	94.1 (4.6)	96.9 (5.8)
Body fat percentage, %	28.7 (4.1)	26.2 (4.3)	27.0 (3.7)	28.1 (3.6)
VO_2_max, ml·kg·min^−1^	41.2 (17.3)	40.6 (16.7)	38.9 (10.5)	37.8 (13.6)
EE during exercise, Kcal	–	462.6 (74.9)	460.9 (86.7)	461.7 (59.1)
Bicep screw curl, 1RM	25.6 (11.6)	23.4 (7.9)	21.9 (10.3)	20.9 (6.9)
Triceps extension, 1RM	16.1 (5.7)	16.1 (5.3)	17.1 (4.9)	17.9 (4.0)
Dumbbell side lateral, 1RM	9.0 (1.9)	10.8 (3.6)	8.9 (1.9)	10.2 (3.3)
Military press, 1RM	25.8 (9.4)	23.0 (8.3)	22.8 (13.5)	19.0 (5.1)
Dumbbell squat, 1RM	47.8 (23.0)	55.0 (34.4)	53.3 (14.1)	52.8 (23.5)
Dumbbell front lunge, 1RM	28.4 (7. 7)	28.0 (16.2)	22.7 (6.6)	26.3 (10.7)
Total muscle strength, (kg; total of six exercises)	142.7 (43.7)	156.3 (53.4)	136.5 (34.6)	147.2 (38.5)
BDNF, ng/mL^*^	176.8 (37.6)	161.1 (24.7)	166.0 (30.5)	189.1 (27.0)
NT-3, ng/mL^*^	247.5 (20.5)	285.4 (41.5)	315.1 (28.6)	275.3 (38.3)
NT-4/5, ng/mL^*^	14.3 (1.7)	18.0 (1.7)	16.4 (1.8)	17.4 (1.7)

Data in mean (standard deviation) or (SEM)^*^. HIIT, high-intensity interval training; RT, resistant training; BMI, body mass index; EE, energy expenditure; VO_2_max, cardiorespiratory fitness; 1RM, one repetition maximal; BDNF, brain-derived neurotrophic factor; NT-3, neurotrophin-3; NT-4/5, neurotrophin-4/5; (–), not applicable.

The concentration of BDNF, NT-3, and NT-4/5 at rest and immediately after the exercise, are presented in Table 3. The variables analyzed were similar at baseline. RT induced significant increases in NT-3 (+39.6 ng/mL [95% CI, 2.5–76.6; *p* = 0.004], and NT-4/5 (+1.3 ng/mL [95% CI, 0.3–2.3; *p* = 0.014]), respectively. Additionally, combined training results in favorable effects on BDNF (+22.0, 95% CI, 2.6–41.5; *p* = 0.029) and NT-3 (+32.9 ng/mL [95% CI, 12.3–53.4; *p* = 0.004]), respectively. In the per-protocol analyses, neither intervention significantly changed BDNF [*F*_(interaction)_ = 1.412; η^2^ = 0.083], NT-3 [*F*_(interaction)_ = 1.280; η^2^ = 0.076], and NT-4/5 [*F*_(interaction)_ = 0.649; η^2^ = 0.040].

**Table 3:** Intent-to-treat analysis of BDNF, NT-3, and NT-4/5 at baseline and changes after acute effect.

**Characteristics**	**Mean (SEM**		**Δ change (*P* value)**	**From baseline to acute, Mean (95% CI)**		**F _interaction_ (η^2^ partial)**
	**Baseline**	**Acute**		**Within-group change**	**Between-group difference in change**	
**BDNF, ng/mL**						
HIIT training, (*n* = 14)	161.0 (23.1)	172.1 (25)	+6.8 (0.134)	11.05 (3.8 to 26.0)	–	–
RT training, (*n* = 12)	166.0 (30.6)	181.5 (31.2)	+9.3 (0.066)	15.5 (−1.2 to 32.3)	–	–
Combined training, (*n* = 13)	189.1 (29.7)	211.2 (34)	+11.6 (0.029)	22.0 (2.6 to 41.5)	–	–
Control group, (*n* = 12)	176.7 (38.7)	177.9 (38.4)	+0.6 (0.804)	1.19 (−11.6 to 9.2)	–	–
Δ Combined vs. Δ control	–	–	–	–	20.8 (−0.1 to 41.8)	1.412 (0.083)
Δ HIIT vs. Δ control	–	–	–	–	9.8 (−10.7 to 30.4)	
Δ RT vs. Δ control	–	–	–	–	14.3 (−35.7 to 7.0)	
Δ Combined vs Δ HIIT	–	–	–	–	11.0 (−9.1 to 31.2)	
Δ RT vs. Δ HIIT	–	–	–	–	4.4 (−16.1 to 25.0)	
Δ Combined vs. Δ RT	–	–	–	–	6.5 (−14.4 to 27.5)	
**NT-3, ng/mL**						
HIIT training, (*n* = 14)	285.3 (46.3)	322.4 (41.5)	+12.5 (0.097)	37.4 (−7.7 to 81.7)	–	–
RT training, (*n* = 12)	315.1 (28.6)	354.6 (28.6)	+13.0 (0.038)	39.6 (2.5 to 76.6)	–	–
Combined training, (*n* = 13)	275.2 (33.7)	308.1 (38.9)	+11.9 (0.004)	32.9 (12.3 to 53.4)	–	–
Control group, (*n* = 12)	247.4 (26.4)	250.0 (25.3)	+1.0 (0.638)	2.5 (−8.9 to 14.0)	–	–
Δ Combined vs Δ control	–	–	–	–	30.4 (−12.4 to 73.2)	1.280 (0.076)
Δ HIIT vs Δ control	–	–	–	–	34.5 (−7.5 to 76.5)	
Δ RT vs Δ control	–	–	–	–	37.0 (−6.5 to 80.7)	
Δ Combined vs Δ HIIT	–	–	–	–	−4.1 (−45.3 to 37.0)	
Δ RT vs Δ HIIT	–	–	–	–	2.5 (−39.5 to 44.6)	
Δ Combined vs Δ RT	–	–	–	–	−6.6 (−49.5 to 36.1)	
**NT**−**4/5, ng/mL**						
HIIT training, (*n* = 14)	17.9 (2.56)	18 (2.32)	+0.5 (0.870)	0.1 (−1.5 to 1.8)	–	–
RT training, (*n* = 12)	16.4 (2.0)	17.5 (2.4)	+6.7 (0.014)	1.3 (0.3 to 2.3)	–	–
Combined training, (*n* = 13)	17.3 (1.9)	18.9 (2.5)	+9.2 (0.246)	1.5 (−1.2 to 4.3)	–	–
Control group, (*n* = 12)	14.2 (2.3)	14.8 (2.5)	+4.2 (0.175)	−0.6 (−0.3 to 1.5)	–	–
Δ Combined vs Δ control	–	–	–	–	0.9 (−1.4 to 3.3)	0.649 (0.040)
Δ HIIT vs Δ control	–	–	–	–	−0.4 (−2.8 to 1.9)	
Δ RT vs Δ control	–	–	–	–	0.7 (−1.7 to 3.2)	
Δ Combined vs Δ HIIT	–	–	–	–	1.4 (−0.8 to 3.7)	
Δ RT vs Δ HIIT	–	–	–	–	1.2 (−1.1 to 3.5)	
Δ Combined vs Δ RT	–	–	–	–	0.2 (−2.1 to 2.6)	

*Data in standard error of the mean (SEM) or (95% CI). HIIT, high–intensity interval training; RT, resistant training; BDNF, brain–derived neurotrophic factor; NT-3, neurotrophin-3; NT-4/5, neurotrophin-4/5; (–), not applicable; Δ, within–group change. η^2^ partial are interpreted as small (≥0.02), moderate (≥0.13), and large (≥0.26)*.

**Figure 3:**
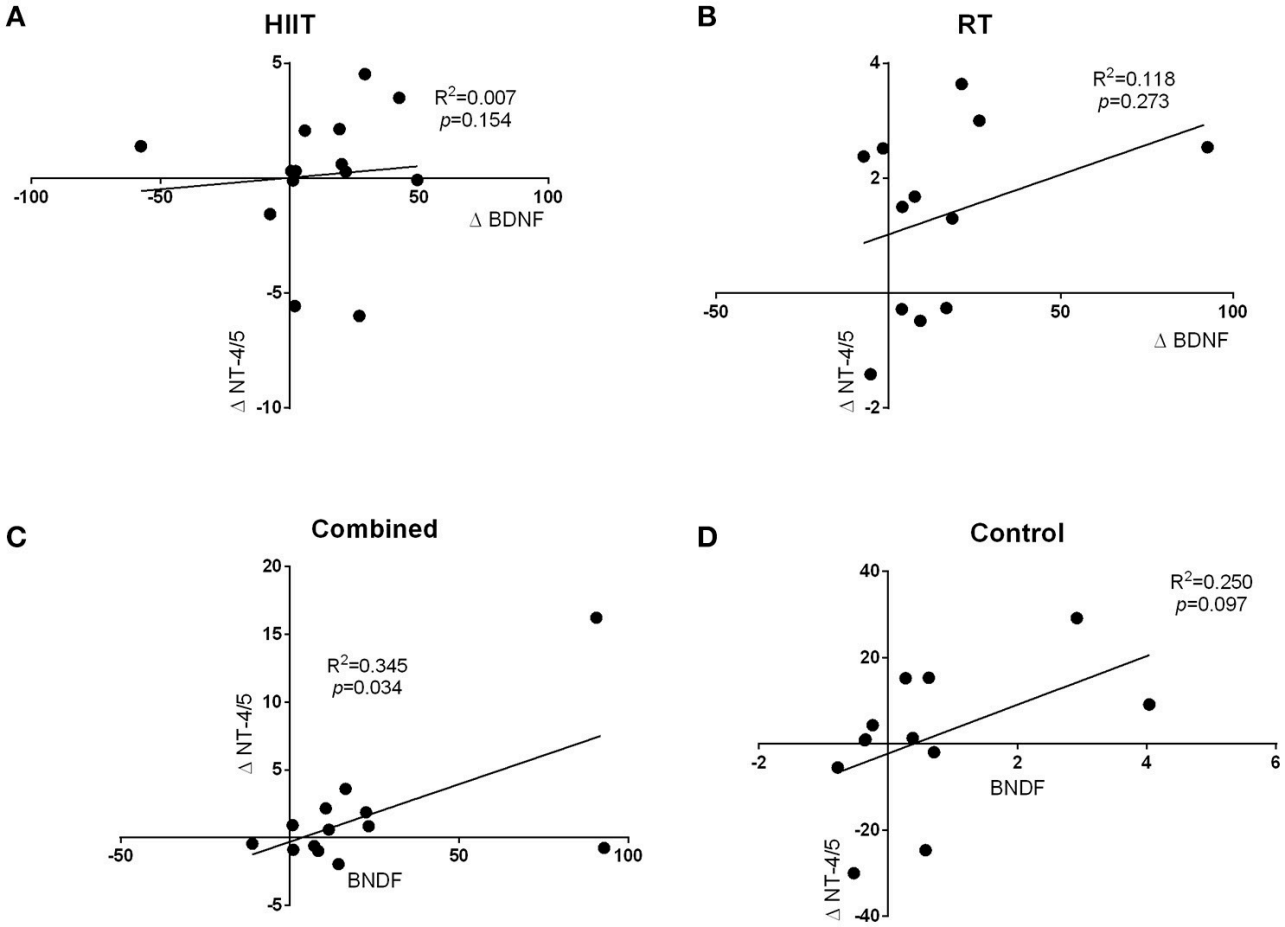
The regression analysis revealed a significant positive relationship between changes in BDNF levels and changes in NT-4/5 levels from baseline to immediate post-exercise in the combined training group (*R*^2^ = 0.345, *p* = 0.034; **C**). Additionally, no relationship was found between changes in BDNF levels and changes in NT-3 levels **(A,B,D)**.

The regression analysis revealed a significant positive relationship between changes in BDNF levels and changes in NT-4/5 levels from baseline to immediate post-exercise in the combined training group (*R*^2^ = 0.345, *p* = 0.034; Figure 3C) but not the other intervention groups. Additionally, no relationship was found between changes in BDNF levels and changes in NT-3 levels (Figures 3A,B,D).

## Discussion

The primary findings were that all three exercise protocols induced greater changes in neurotrophin levels compared to the levels in the baseline levels; however, the combined and RT protocols exhibited greater changes in BDNF, NT-3, and NT-4/5 than the HIIT group. Additionally, we observed a positive relationship between changes in the BDNF levels and changes in the NT-4/5 levels from baseline to immediately post-exercise in the combined training group. These data provide preliminary evidence regarding the role of acute exercise in increasing neurotrophic factors in humans (Krabbe et al., 2007; Rasmussen et al., 2009; Seifert et al., 2010; Saucedo-Marquez et al., 2015) and confirm the results observed in mice (Gómez-Pinilla et al., 2001; Johnson and Mitchell, 2003; Chung et al., 2013). In addition, it also suggests that an concurrent training protocol (high intensity intermittent exercise followed by strength training) may be more suitable for influencing neurotrophin levels than HIIT or RT alone.

Within the training protocols, we observed an 6.8% (Combined training), and 9.3% (RT) mean increase in plasma BDNF levels immediately post-exercise when compared with the baseline values. Studies that analyzed the acute effects of RT (Correia et al., 2010; Goekint et al., 2010; Yarrow et al., 2010; Church et al., 2016) or HIIT (Saucedo-Marquez et al., 2015; Tonoli et al., 2015) showed inconsistent results. Our RT protocol comprised exercises at an intensity between 50 and 70% of 1RM with 25–30 repetitions per exercise. Compared to the aerobic protocols, the RT session had relatively large resting periods in between the efforts. By contrast with our results, several studies demonstrated that a single RT session did not induce significant changes in blood BDNF levels in healthy inactive adults (Goekint et al., 2010; Yarrow et al., 2010). However, Church et al. (2016) and Marston et al. (2017) reported that the use of a to-fatigue hypertrophy-based RT protocol provides the necessary stimulus to increase peripheral serum BDNF levels.

The discrepancies observed between our results and these findings could be due to the population sampled since BDNF levels are negatively correlated to weight (Lommatzsch et al., 2005); therefore, the probability of increases could be greater in the overweight population included in our study. Regarding high-intensity exercise or combined exercise, a previous study in 10 inactive individuals with type 1 diabetes showed that acute HIIT protocols result in larger increases in serum BDNF concentrations than acute low- or moderate-intensity exercise protocols (Tonoli et al., 2015). Therefore, these studies have shown that various features of exercise stimuli (i.e., intensity, duration, and mode of activity) can affect BDNF levels. It seems that exercise and/or training temporarily elevate basal BDNF levels and possibly upregulate the cellular processing of this factor (Knaepen et al., 2010). In future studies, it could be interesting to investigate the effects of an intensive strength protocol on BDNF levels.

Of particular interest, we observed that changes in BDNF were significantly related to changes in NT-4/5 levels from baseline to immediately post-exercise in the combined training group. In literature, the %Δ [BDNF] concentrations after performing exercise vary from 11.7 to 41% (in both healthy and clinical populations) (Pedersen et al., 2009; Yarrow et al., 2010; Marston et al., 2017). These results are very much in line with results reported by Saucedo-Marquez et al. (2015), who showed that BDNF levels gradually increased over time, reaching maximal levels after 20 min of exercise and returned to baseline values after 10 min of recovery. However, this finding was also present in animal studies showing positive relationships between serum hippocampal NT-3 concentration and distance ran (*R*^2^ = 0.157, *p* < 0.001) (Johnson and Mitchell, 2003). Similarly, Chung et al. (2013) suggested that NT-4/5 levels were altered in response to ischemic injury, and treadmill exercise plays a role in the changes of the levels of neurotrophins and their receptors. However, treadmill exercise-mediated changes in the expression levels of NT-4/5 and tyrosine kinase B might participate in the recovery process in rats with brain damage. This supports the idea that increasing the levels of neurotrophic factors contributes to functional recovery (Keyvani et al., 2004).

The potential mechanism mediating the higher neurotrophin (i.e., BDNF) response to the different exercise protocols is currently unclear. However, BDNF is primarily produced in the brain, some of which crosses the blood-brain barrier and travels to the periphery where it can be measured in plasma and serum. Nevertheless, possible BDNF modulating factors such as lactate, cortisol, and intensity have been previously proposed. Wrann et al. (2013) proposed a novel biochemical pathway linking an exercise-induced secreted factor from skeletal muscle to BDNF gene expression in the brain, especially in the hippocampus. Interestingly, then RT or concurrent training protocol (HIIT followed by RT) would be the ideal protocol for elevating BDNF levels. On the basis of this model we speculate that skeletal muscle contractions during combined or RT protocol might be a possible trigger of this biochemical pathway to induce elevated BDNF levels in the brain. Another physiological mechanism is platelets, which have the ability to store BDNF and release it upon agonist stimulation depending on the specific need of BDNF in certain tissues (Fujimura et al., 2002). Although it remains unknown how exercise influences the platelets, one potential use of BDNF stored in platelets is thought to be in the repair of exercise-induced muscle damage (Saucedo-Marquez et al., 2015). However, further research is needed to confirm these mechanisms, especially in childhood obesity during and after weight-loss exercise programs.

On the other hand, several lines of evidence support a hypothesis whereby neurotrophins, especially BDNF (Dmitrzak-Weglarz et al., 2013), play an essential role in with the initiation and development of both atherosclerosis and metabolic syndrome (Chaldakov et al., 2003). In a previous study Krabbe et al. (2009) found that plasma levels of BDNF decreased in humans with type 2 diabetes, independently of obesity, suggesting that certain metabotrophic deficit due to hyponeurotrophinemia may operate in this metabopathy. Matthews et al. (2009) have shown that BDNF appears to be a major player not only in central metabolic pathways, but also as a regulator of metabolism in skeletal muscles. Studies of animal models and humans have shown that reduced expression of neurotrophins results in the glucose and lipid metabolism alterations (Tonra et al., 1999; Nonomura et al., 2001; Mercader et al., 2008; Dmitrzak-Weglarz et al., 2013). In general, studies have observed a correlation between low serum BDNF levels and high inflammatory protein levels, increased vascular dysfunction and more cardiovascular risk factors (Chaldakov et al., 2004; Dmitrzak-Weglarz et al., 2013). Current evidence indicates plasma BDNF is a proxy marker of BDNF production in the brain; however, BDNF can also be synthesized peripherally (Church et al., 2016). However, there are some gaps in the literature pertaining to obesity, exercise training, and subsequent neurotrophin responses (Pedersen et al., 2009).

There are several limitations and strengths that should be considered with respect to the design and outcomes of this study. Because this study was designed as a pilot study to inform the development of more elaborate trials, the sample size, and intervention duration of acute exercise are modest. This study was performed with physically inactive individuals, which could complicate the generalization of results to a more active overweight population; also, the study was performed with volunteers, which could lead to sampling bias. While our results argue that this level of physical activity is feasible for many, it remains to be seen whether the reported duration is the most efficacious.

To our knowledge, this is the first study to examine whether HIIT, RT, or HIIT+RT elicit different effects on plasma levels of NT-3, NT-4/5, and BDNF in inactive overweight adults. Interestingly, despite this evidence, there are few clinical trials that have directly evaluated the effects of sustained exercise regimens on the brain health of inactive adults (Saucedo-Marquez et al., 2015; Church et al., 2016; Barha et al., 2017; Dinoff et al., 2017). The strategy immobilized antibodies onto sensor surfaces for quantification of protein using biosensors, which is more accurate and fast compared to traditional assays (Heinrich et al., 2010; Wöllner et al., 2010; Hu et al., 2013; Tam et al., 2017). The determination of levels of neurotrophins is usually performed by ELISA immunoassays; however, the use of biosensors opens a new possibility in obtaining results in real time, label free with a high sensitivity and specificity.

## Conclusion

Our findings indicate that the use of several exercise protocols (i.e., fatigue hypertrophy-based RT or combined training) provides the necessary stimuli to increase peripheral plasma neurotrophin levels among inactive overweight individuals. Based on our findings, however, the use of concurrent training may be considered in exercise programs to enhance the possibility of a potential metabotropic benefit for individuals due to increased expression of neurotrophic factors. Additional longitudinal studies should be performed to establish the effects of exercise and training on cognitive function in overweight adults over the long term.

## New and noteworthy

Identifying the training regimen that has the most beneficial effects on each parameter could potentially lead to enhanced precision in prescribing exercise training intensity to achieve optimal outcomes in this population.

The present study demonstrates that that acute resistance training and combined exercise increase neurotrophic factors in physically inactive overweight adults. Not all neurotrophic factors measured responded the same to this type of exercise, suggesting different regulatory mechanisms and time courses for induction.

## Author contributions

MD-S, RB-C, and RR-V conceived and designed the project. EH, GV-O, MD-S, KG-R, and JC-B reviewed the literature studies and conducted data extraction. MD-S and CP-G conducted data analyses. RR-V, HT-R, AG-H, and MI were responsible for data interpretation. RB-C, AQ, CP-G AT-S, MD-S, MI, and RR-V drafted the manuscript. JP-I, LT-T, and AT-S revised it critically for intellectual contributions. MD-S, RB-C, and RR-V coordinate the study development. All authors reviewed and edited the manuscript. All authors read and approved the final manuscript.

### Conflict of interest statement

The authors declare that the research was conducted in the absence of any commercial or financial relationships that could be construed as a potential conflict of interest.

## Abbreviations

BDNF: brain-derived neurotrophic factor
neurotrophin-3: NT-3
NT-4/5: neurotrophin-4/5
HIIT: high-intensity interval training
GDNF: glial cell line-derived neurotrophic factor
TNF-α: tumor necrosis factor alpha
CEMA: in Spanish at the Centre of Studies in Physical Activity Measurements
RT: resistance training
WHO: world health organization
HR: heart rate
1RM: one repetition maximum
BMI: body mass index
TEM: technical error of measurement
BIA: bioelectrical impedance analyser
VO2max: maximal oxygen intake.
